# A correlation-based tool for quantifying membrane periodic skeleton associated periodicity

**DOI:** 10.3389/fninf.2025.1628538

**Published:** 2025-08-22

**Authors:** Sam K. Vanspauwen, Virginia Luque-Fernández, Hanne B. Rasmussen

**Affiliations:** Department of Biomedical Sciences, Faculty of Health and Medical Sciences, University of Copenhagen, Copenhagen, Denmark

**Keywords:** membrane-associated periodic skeleton, super-resolution microscopy, autocorrelation, cross-correlation, axon initial segment, napari, software

## Abstract

**Introduction:**

The advent of super-resolution microscopy revealed the membrane-associated periodic skeleton (MPS), a specialized neuronal cytoskeletal structure composed of actin rings spaced 190 nm apart by two spectrin dimers. While numerous ion channels, cell adhesion molecules, and signaling proteins have been shown to associate with the MPS, tools for accurate and unbiased quantification of their periodic localization remain scarce.

**Methods:**

We developed Napari-WaveBreaker (https://github.com/SamKVs/napari-k2-WaveBreaker), an open-source plugin for the Napari image viewer. The tool quantifies MPS periodicity using autocorrelation and assesses periodic co-distribution between targets using cross-correlation. Performance was evaluated using both simulated datasets and STED microscopy images of periodic and non-periodic axonal proteins.

**Results:**

Napari-WaveBreaker output parameters accurately reflected the visually observed periodicity and detected spatial shifts between two periodic targets. The approach was robust across varying image qualities and reliably distinguished periodic from non-periodic protein distributions.

**Discussion:**

Napari-WaveBreaker provides an unbiased, quantitative framework for analyzing MPS-associated periodicity and co-distribution enabling new insights into the molecular organization and modulation of the MPS.

## 1 Introduction

The spectrin-actin membrane skeleton, initially unveiled in the context of erythrocyte biology, stands as an integral structure in cellular research. Early research exploring the composition of the structure laid the foundation for understanding its evolutionary importance and its role in cell polarization, adhesion, stress resistance, and general structural integrity (Baines, 2010). First imaged by Byers and Branton (1985) using electron microscopy, the structure consists of 200 nm long spectrin tetramers crosslinked by actin filaments, of which the binding depends on the presence of protein 4.1 (Ungewickell et al., 1979; Fowler and Taylor, 1980; Tyler et al., 1980) and adducin (Gardner and Bennett, 1987). Another key player, ankyrin, is required for the membrane association of the complex (Bennett and Stenbuck, 1979, 1980).

While neurons were known to express a spectrin-actin membrane skeleton (Bennett et al., 1982), it was the development of advanced superresolution light microscopy techniques such as stimulated emission depletion microscopy (STED) and stochastic optical reconstruction microscopy (STORM) that facilitated a surge in the studies on the organization of the neuronal cytoskeletal complex. Using STORM, Xu et al. (2013) reported the periodic distribution of actin in ring-like structures along neuronal axons, the rings being interconnected by spectrin tetramers. The structure has since gained the name membrane-associated periodic skeleton (MPS, He et al., 2016). Since its original discovery, similar structures have been described in subregions of dendrites, in glial cells, and in various species (He et al., 2016). Furthermore, in line with the inherent periodicity of the axonal cytoskeletal structure, numerous axonal ion channels have been shown to exhibit similar periodic localization patterns, either co-localizing with the actin rings or localizing in-between them (D'Este et al., 2015, 2016). When Xu et al. (2013) revealed the axonal MPS, they showed that voltage-gated sodium channels (Navs) in the axon initial segment (AIS) exhibited a periodic localization corresponding to that of ankyrin G (ankG), corroborating the known interaction between the two proteins (Zhou et al., 1998; Lemaillet et al., 2003). More recently, the mechanosensitive potassium channel TRAAK was shown to display a similar ankG-dependent periodic localization (Luque-Fernández et al., 2024). In contrast, the voltage-gated potassium channel Kv1.2 was shown to be periodically co-localized with the actin rings of the AIS, as was the voltage-gated potassium channel Kv7.2 in nodes of Ranvier (D'Este et al., 2017). Intriguingly, it is not only ion channels that exhibit such periodic organizations as a number of cell adhesion molecules, motor proteins, and signaling molecules have also been demonstrated to display periodic localizations that appear to follow the structure of the MPS (D'Este et al., 2015; Berger et al., 2018; Abouelezz et al., 2020; Zhang et al., 2023).

The discovery of the MPS and its associated proteins has created a demand for reliable quantification of the relative level of periodicity. This would allow quantitative comparisons of the periodic localization of, for instance, wildtype and mutant proteins (Hefting et al., 2020; Luque-Fernández et al., 2024) or determining whether the periodicity of a protein is affected by a treatment or experimental setup like genetic knockout or shRNA knockdown of MPS proteins (Zhou et al., 2022). Demonstrating periodic localization is conventionally done by showing the fluorescence intensity profile of a drawn line or selected image region perpendicular to the periodicity (Berger et al., 2018), sometimes accompanied by a fitted sinusoidal curve (Leterrier et al., 2015) or the autocorrelation profile (Albrecht et al., 2016; D'Este et al., 2017; Hauser et al., 2018; Abouelezz et al., 2020; Fréal et al., 2023). In addition, a value that indicates the periodicity level is often extracted, for example, using the goodness of fit for the fitted sinusoidal curve (Leterrier et al., 2015) or using local minima and maxima of the autocorrelation profile (Zhong et al., 2014; He et al., 2016; Han et al., 2017; Wang et al., 2019; Vassilopoulos et al., 2019; Zhou et al., 2022). However, one key issue with the mentioned approaches is that they are based on a manually selected region, which unavoidably introduces bias. A method by Barabas et al. (2017) addressed this issue by splitting super-resolution images into multiple segments that were analyzed individually, then comparing the image with a generated periodic pattern with a frequency of 190 nm using two-dimensional Pearson correlation. Correlation surpassing a pre-determined threshold was deemed periodic in the segment. Notably, the technique was built with user experience and accessibility in mind. While this method was less biased, it introduced other issues like a limiting pattern frequency of precisely 190 nm and the use of Pearson correlation, which is sensitive to background noise. Furthermore, a pre-determined correlation threshold may not be universally applicable, as it does not provide much flexibility for periodic variability between samples or in the same sample where periodic patterns are only partially present. More recently, the Structural Repetition Detector (SReD) was introduced as a general framework for detecting structural repetitions in microscopy images, including ring-like patterns such as those seen in axonal spectrin arrangements. SReD compares local blocks within an image to identify repeated structures, operating without prior assumptions about pattern scale or orientation (Mendes et al., 2025). While SReD offers high sensitivity and broad applicability, it relies on correlation with reference blocks and generates structural repetition maps. An associated pattern prominence metric reflects the relative strength of detected repetition. However, because this metric is derived from block-wise similarity rather than directly from signal intensity profiles, it may be less intuitive to interpret in the context of conventional periodicity analysis. Additionally, none of the above-mentioned tools allow for the analysis of two periodic protein co-distribution, essential to unravel the organization of the MPS.

In this paper, we introduce a newly developed, publicly available analysis tool, Napari-WaveBreaker (https://github.com/SamKVs/napari-k2-WaveBreaker), that takes advantage of the unbiased and accessible approach of Barabas et al. (2017) and the flexibility and accuracy of autocorrelation. Furthermore, we apply cross-correlation analysis to determine the spatial shift between two periodic patterns, allowing for estimations of whether two proteins co-distribute or alternate in their periodic localizations.

## 2 Methods

**Figure 1:**
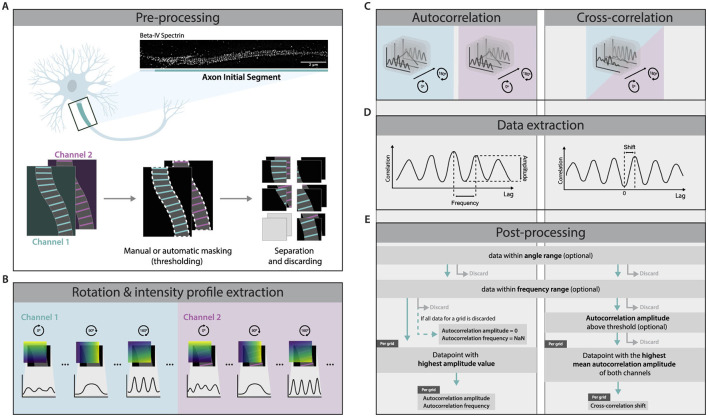
Method Overview. **(A)** Super-resolution images were masked by automatic thresholding, manual roi selection by drawing, or a combination of the two. Then, the images were separated into multiple grids (grid size and split method determined by the user). When using two-channel images, the same mask and separation were applied to both. If a grid contained no masked area, it was discarded. **(B)** For each channel and grid, intensity profiles were extracted over multiple angles. **(C)** Autocorrelation was performed on each extracted intensity profile. Cross-correlation was performed using the intensity profiles of both channels for each respective degree. **(D)** Frequency and amplitude were extracted from the autocorrelation profile. The frequency was defined as the lag of the second maximum of the profile and amplitude was quantified as the difference between the autocorrelation values at the second maximum and the first minimum. The cross-correlation shift was determined as the lag of the maximum with the highest correlation value among the two maxima that were closest to lag zero. **(E)** Datapoints outside of a predefined angle and frequency range were discarded. For autocorrelation, the data point with the highest amplitude value was kept, and the autocorrelation amplitude and frequency were extracted. For cross-correlation, the data point with the highest average autocorrelation amplitude of both channels was kept, and the cross-correlation shift was extracted.

The following workflow aims to quantify super-resolution images containing MPS-like structures in an unbiased manner. It consists of several steps, including preprocessing, extraction of intensity profiles, correlation, and finally, data extraction and post-processing (schematically summarized in Figure 1). The analysis can be done on single-channel and two-channel images. For single-channel images, the sequence of computations provides the autocorrelation amplitude at a specific frequency, which represents the level of periodicity. In the case of dual-channel images, the frequencies and autocorrelation amplitudes are extracted from both channels in addition to the periodic shift between the channels as provided by cross-correlation. The method will be described assuming full correlation analysis, meaning autocorrelation analysis on two channels and cross-correlation analysis between the channels.

### 2.1 Algorithm

#### 2.1.1 Preprocessing

Raw images were first preprocessed to define the region of interest and prepare the image for further processing (Figure 1A). Discrimination from the background was done by manual thresholding and smoothing on the channel with the best contrast between signal and background. In the case of unwanted artifacts, the binary mask was manually adjusted by drawing or erasing certain areas. To avoid selection bias, the final mask encompassed the full axon present in the image. In our experimental setup, elements that justified the exclusion of certain regions were partially out-of-focus areas or other neurites running over or under the axon of interest. Hereafter, the image was divided into multiple grids of a user-defined width and height. For our experiments, the grid width was set to match the width of the masked image, as the captured axons were relatively straight and linear. Furthermore, consistent with Barabas et al. (2017), we found that a height of 1 μm was most appropriate due to the 190 nm periodic interdistance of the MPS, the non-linear nature of axons, and possible variations in periodicity along the axon. Grids containing only background were excluded from further processing.

#### 2.1.2 Intensity profile extraction

To find the angle at which an individual grid exhibits the best periodicity, analysis of intensity profiles at multiple angles is required. Additionally, the analysis can be defined to be unrestricted or restricted to a user-defined angle range. To extract intensity profiles from a grid at multiple angles, gradient-like label layers were generated for each angle (Figure 1B). These label layers were created by drawing lines perpendicular to the angle of interest, using Bresenham's line algorithm (Bresenham, 1965), with each line assigned a unique integer label. The lines were then shifted across the entire image, each with a subsequent label, ensuring full coverage and forming a gradient-like structure. For each label, the corresponding pixels' intensity values were averaged, and the resulting intensity profile was plotted as a function of the label values across the image.

#### 2.1.3 Correlation

Before autocorrelation analysis, intensity profiles were normalized to center the autocorrelation profile around a correlation value of 0. This normalization was achieved by subtracting the mean value of the profile from each data point, resulting in a normalized profile *n*′. The autocorrelation profile was then computed for each normalized profile to evaluate the self-similarity across different lag values *k*.

The autocorrelation function *A*(*k*) was defined as follows:


(1) $$\begin{array}{l} A\left(k\right)=\overset{N-k}{\underset{i=1}{\sum }}n_{i}^{\prime }\cdot n_{i+k}^{\prime }\;\text{for }k=0\text{ to }\left(N-1\right), \end{array}$$

where $n_{i}^{\prime }$ represents the normalized intensity at position *i*, and *N* is the total number of points in the profile.

The autocorrelation profile *A*(*k*) was then further normalized to ensure the autocorrelation at lag value 0 is 1, which facilitates comparisons between different profiles. The normalized autocorrelation for each lag *k* was obtained by:


(2) $$\begin{array}{l} A_{\text{norm}}\left(k\right)=\frac{A\left(k\right)}{N\cdot \sigma^{2}} \end{array}$$

In this equation, *A*_norm_(*k*) denotes the normalized autocorrelation at lag *k*, *A*(*k*) represents the raw autocorrelation value obtained previously, *N* is the total number of points in the normalized intensity profile, and σ is the standard deviation of the normalized intensity values.

Finally, cross-correlation was performed using the corresponding intensity profiles from both channels.

The cross-correlation function *C*(*k*) was defined as follows:


(3) $$\begin{array}{l} C\left(k\right)=\overset{N-k}{\underset{i=1}{\sum }}n_{1,i}^{\prime }\cdot n_{2,i+k}^{\prime }\;\text{for }k=0\text{ to }\left(N-1\right), \end{array}$$

where $n_{1,i}^{\prime }$ and $n_{2,i}^{\prime }$ represent the normalized intensity at position *i* of channels 1 and 2 respectively. *N* is the total number of points in the profile.

The cross-correlation profile *C*(*k*) was then further normalized. The normalized cross-correlation for each lag *k* was obtained by:


(4) $$\begin{array}{l} C_{\text{norm}}\left(k\right)=\frac{C\left(k\right)}{N\cdot \sigma_{1}\cdot \sigma_{2}} \end{array}$$

In this equation, *C*_norm_(*k*) denotes the normalized autocorrelation at lag *k*, *C*(*k*) represents the raw cross-correlation value obtained previously, *N* is the total number of points in the normalized intensity profile, and σ_1_ and σ_2_ are the standard deviations of the normalized intensity values for channel 1 and channel 2 respectively.

#### 2.1.4 Data extraction

From the autocorrelation profiles, two parameters were extracted: amplitude and frequency (Figure 1D). Local minima and maxima were defined by evaluating the discrete derivative of the autocorrelation profile. The frequency of the autocorrelation was defined as the lag of the second maximum of the profile. Conversely, amplitude was quantified as the difference between the autocorrelation values at the second maximum and the first minimum.

In the case of cross-correlation, analogous to the procedure in autocorrelation, the discrete derivative of the cross-correlation profile was first computed. The derivative was utilized to locate the local maxima. Subsequently, the cross-correlation shift was determined as the lag of the maximum with the highest correlation value among the two maxima that are closest to lag zero.

#### 2.1.5 Post-processing

Following the autocorrelation analysis, each grid yielded frequency and amplitude values for all considered angles. The analysis can be defined by the user to be unrestricted or restricted to an angle range and/or frequency range during the post-processing.

For autocorrelation, the data was further filtered by retaining only the data point corresponding to the highest autocorrelation amplitude per grid, resulting in a single autocorrelation amplitude and corresponding frequency per analyzed grid. If all data points for a grid were discarded due to angle and frequency filtering, that grid was assigned a NaN frequency value and an autocorrelation amplitude of 0.

For cross-correlation analysis, only the angle with the highest average autocorrelation amplitude between both channels was retained for each grid, and the cross-correlation shift corresponding to this angle was used as the resulting cross-correlation shift. Optionally, the data can be subjected to an autocorrelation amplitude threshold on both channels. In this case, grids for which all angles were discarded would be excluded from further analysis.

To further investigate relative positioning between targets, the cross-correlation shift for each remaining grid and angle was normalized by first calculating:


(5) $$\begin{array}{l} L=\frac{F_{1}+F_{2}}{4} \end{array}$$

where *L* is defined as the midway point between the average of the autocorrelation frequencies from two channels, *F*_2_ and *F*_2_ respectively.

The normalized cross-correlation shift was then calculated by:


(6) $$\begin{array}{l} S^{\prime }=L-|\left(S\text{ mod }\left(2L\right)\right)-L| \end{array}$$

where *S* and *S*′ refer to the cross-correlation shift and normalized cross-correlation shift, respectively. Furthermore, each normalized shift was classified as overlapping or alternating via:


(7) $$\text{Call}(S^{\prime })=\{\begin{array}{ll} \text{"Overlapping"}, & \text{if }S^{\prime }<\frac{L}{2}\\ \text{"Alternating"}, & \text{if }S^{\prime }\geq \frac{L}{2} \end{array}$$

### 2.2 Napari plugin

The methodology was designed with a focus on accessibility. Therefore, the method was packaged as a user-friendly plugin for Napari (RRID:SCR_022765), a Python-based image analysis tool that has gained substantial popularity in recent years (Sofroniew et al., 2022). The tool was named Napari-WaveBreaker (RRID:SCR_027179). The plugin enables control over the desired analysis angles and grid dimensions. Additionally, it allows automated masking and manual mask editing. Image processing is automated in the plugin up to the data extraction phase. Post-processing was not included in the plugin to facilitate various approaches for data handling. However, the described approach is available in the form of Excel templates and Python scripts. This plugin is publicly available on GitHub (https://github.com/SamKVs/napari-k2-WaveBreaker) and Napari Hub. All analyses in this study were performed using Napari-WaveBreaker version 0.2.2 (Vanspauwen, 2023).

### 2.3 Validation

The method was validated in two ways. It was first applied to simulated images, which allowed control over parameters that can potentially influence the results. It was subsequently applied to STED microscopy images of AIS localized targets.

#### 2.3.1 Image simulation

To validate the methodology and maintain full control over the parameters influencing the resulting autocorrelation amplitude and cross-correlation shift, we simulated images resembling STED images of the MPS. Simulated images were generated with all possible values from the parameters listed in Table 1. For each parameter, all possible values were simulated, while the remaining parameters were held constant at their default values (highlighted in bold in Table 1). The default values were chosen based on resemblance to the STED images being mimicked. The RED kernel simulates the Abberior STAR RED fluorophore, which offers a higher resolution than the STAR ORANGE fluorophore, so it was preferentially used when imaging single-channel immunolabelings. The STED images were acquired at a resolution of 100 pixels/μm, and deconvolution of the images (see more below) resulted in noise-free images with low signal variance. Thus, no noise and 100% signal variance were the chosen default parameters. Finally, 100 points/μm^2^ was the average point density obtained in STED images under our experimental conditions.

**Table 1:** Simulation parameters and their respective possible values.

**Parameters**	**Possible values**
Kernel	**RED**, ORANGE
Noise	**None**, 5%, 10%, 20%, 30%
Pixel size	50, **100** (pixels/μm)
Point density	25, 50, **100**, 200, 300 (points/μm^2^)
Signal variance	30-100%, **100%**

Values highlighted in bold are referred to as default.

**Figure 2:**
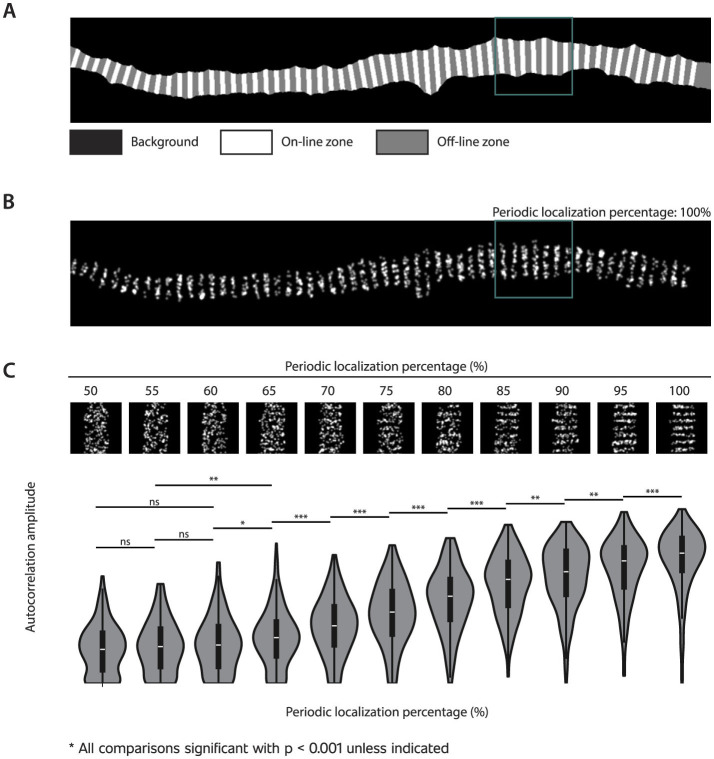
Autocorrelation analysis of simulated images. **(A)** Simulated images were based on masks of STED AIS images of cultured rat hippocampal neurons, with the black area representing the background. The remaining region was divided into zones: white, which depicts the on-line zones spaced 200 nm apart, and gray, which depicts the off-line zones. **(B)** Points were generated in both the on-line and off-line zones with varying periodic localization percentages. In this case, the generated periodic localization percentage is 100%, as all points are located in the on-line zones. The green square indicates an example of an analyzed grid. **(C)** Examples of grids with the indicated periodic localization percentages and the resulting autocorrelation amplitudes for all analyzed grids (n = 195 per periodic localization percentage). Statistical significance was determined using pairwise Mann-Whitney U tests (***for *p*-value < 0.001, **for *p*-value < 0.01, *for *p*-value < 0.05, and “ns” for non-significant). All non-indicated comparisons were significant, with a *p*-value < 0.001.

Simulations were performed on ten masks from randomly selected AIS STED images of cultured rat hippocampal neurons. These images were captured at 50 or 100 pixels/μm according to the *pixel size* simulation parameter. For each image, the backbone of the mask was extracted using the skeletonization methodology of Lee et al. (1994) and filtering out the longest path. Points were spread on the backbone with an interpoint distance of 200 nm. Lines were generated using the Bresenham (1965) algorithm perpendicular to the angle between each point and its three bidirectional neighbors. These lines were subsequently enlarged using dilation within the mask boundaries. The image was then divided into three zones: background, on-line, and off-line (Figure 2A). Points were spawned in the on-line and off-line zones at various periodic localization percentages to create a range of images with different levels of periodicity. At a periodic localization percentage of 50%, points were scattered randomly across the entire mask, regardless of zone distinctions. In contrast, at a periodic localization percentage of 100%, points were exclusively distributed within the on-line zones, creating a perfectly periodic pattern (Figure 2B). Images were generated at percentage intervals of 5%, ranging from 50% to 100%.

The total number of points (*P*_total_) spawned in the image was determined by a predefined *point density* simulation parameter (*D*). *P*_total_ was calculated as follows:


(8) $$\begin{array}{l} P_{\text{total}}=D\cdot \frac{A_{\text{on-line}}+A_{\text{off-line}}}{PPM^{2}} \end{array}$$

where *A* represents the pixel count of each respective zone, and *PPM*^2^ stands for pixels per μm^2^ and represents the pixel density of the image. *D* represents the predefined simulated point density in points per μm^2^.

The number of points spawned in each zone was calculated as follows:


(9) $$\begin{array}{l} P_{\text{on-line}}=P_{\text{total}}\cdot \frac{A_{\text{on-line}}\cdot F}{A_{\text{on-line}}\cdot F+A_{\text{off-line}}\cdot \left(100-F\right)} \end{array}$$

(10) $$\begin{array}{l} P_{\text{off-line}}=P_{\text{total}}\cdot \frac{A_{\text{off-line}}\cdot \left(100-F\right)}{A_{\text{on-line}}\cdot F+A_{\text{off-line}}\cdot \left(100-F\right)} \end{array}$$

Here, *P* denotes the number of points spawned within the respective zones, *A* represents the area or pixel count of each zone, and *F* signifies the predefined periodic localization percentage.

Hereafter, in an empty image, a number of random pixels according to *P*_on-line_ and *P*_off-line_ were given a value in their respective zones. This value was either 1 or a random value between 0.3 and 1, depending on the *signal variance* simulation parameter. This intermediate image was then convolved with a RED or ORANGE kernel, depending on the *kernel* simulation parameter. RED and ORANGE kernels were created by manually isolating 22 to 27 freestanding fluorescent signal dots from real STED images using Abberior STAR RED and Abberior STAR ORANGE coupled secondary antibodies captured at 50 or 100 pixels/μm. A Gaussian curve was fitted for each isolate and averaged to create a RED and ORANGE kernel for both 50 and 100 pixels/μm. Finally, a Gaussian noise layer was added to the simulated image, with a mean of zero and a standard deviation proportional to the maximum intensity of the signal kernel. Specifically, the standard deviation was set to 5%, 10%, 20%, or 30% of the kernel's maximum intensity, corresponding to the defined *noise* simulation parameter. This noise was applied uniformly across the entire image to simulate background intensity variation.

All generated images were analyzed using the workflow described above, with a grid height of 1 μm and a grid width equal to the image width, a frequency range of 170 nm to 230 nm, and an angle range of -20° to +20°.

For cross-correlation simulation experiments, images were generated using default simulation parameters. At various periodic localization percentages, overlapping channels were created by re-spawning points in the different zones using the same mask and zone map. In contrast, alternating channels were generated by shifting points along the skeleton backbone by 100 nm to create an alternating zone map, allowing points to be spawned in alternating zones. Analysis of all simulated images was done with an automated version of Napari-WaveBreaker v0.2.2 (Vanspauwen, 2023).

#### 2.3.2 STED microscopy of cultured hippocampal neurons

All animals were used according to the guidelines of the Danish Veterinary and Food Administration and the Ministry of Food, Agriculture and Fisheries of Denmark. No specific authorization was required for the euthanization of the animals for subsequent tissue preparation, and the procedure was approved by the Department of Experimental Medicine at the University of Copenhagen. Hippocampal neuronal cultures were prepared as previously reported (Luque-Fernández et al., 2024). Shortly, hippocampi from E18 Wistar rat (Charles River, RRID: RGD_737929) embryos of unknown sex were dissected. For each experimental replicate, all embryos from a single pregnant dam were dissected (12–20 embryos), and all hippocampi were pooled before dissociation. The tissue was enzymatically dissociated in 0.25% trypsin (ThermoFisher Scientific, 15090046), and the neurons were plated on 25 mm diameter glass coverslips (Marienfeld Superior, 0117650), precoated with 50 μg/ml poly-D-lysine (ThermoFisher Scientific, A3890401). The neurons were cultured in dishes containing an astroglial feeder layer in 2% B27, 0.5 mM glutamax, and 10 IU/ml penicillin-streptomycin-supplemented Neurobasal medium (all from ThermoFisher Scientific: 17504001, 35050038, 15140122, 21103049). At 3 DIV, 3 μM cytosine arabinoside (Sigma-Aldrich, C6645) was added to stop the proliferation of non-neuronal cells.

At 22 days *in vitro* (DIV), neurons were fixed in 2% paraformaldehyde in PBS (ThermoFischer Scientific, 15434389) for 2 minutes at room temperature, followed by 10 minutes in methanol (VWR, 20846) at -20 °C. For Triton X-100 extracted samples, neurons were exposed to 0.5% Triton X-100 (Sigma-Aldrich, X100) in PBS for 5 minutes at -4 °C prior to fixation. Then, neurons were blocked for 30 min at room temperature in 0.2% fish skin gelatin (Sigma-Aldrich, G7765) and 0.1% Triton X-100 containing PBS. Hereafter, neurons were immunolabeled with mouse anti-betaIV spectrin (1:100, RRID:AB_2315818, UC Davis/NIH neuroMab Facility, Antibodies Incorporated, 75-377), mouse anti-Kv1.1 (1:100, RRID:AB_2128566, UC Davis/NIH neuroMab Facility, Antibodies Incorporated, 75-105) or mouse anti-Kv2.1 (1:100, RRID:AB_10673392, UC Davis/NIH neuroMab Facility, Antibodies Incorporated, 75-014) antibodies in combination with rabbit anti-ankG antibodies (1:2000, RRID:AB_2661876, SYSY, 386 003). Detection was carried out with secondary antibodies coupled to Abberior STAR ORANGE (1:200, RRID:AB_2847853, Abberior, STORANGE-1001-500UG) or Abberior STAR RED (1:200, RRID:RRID:AB_2833015, Abberior, STRED-1002-500UG). Primary and secondary antibodies were applied at room temperature in blocking solution for 1 h. Coverslips were mounted on microscope slides using ProLong Diamond Antifade mountant (ThermoFisher Scientific, P36970).

Images were acquired with a Zeiss Axio Imager Z1 microscope attached to a STEDYCON STED system (Abberior). A 100×, 1.46 numerical aperture, oil-immersion objective was used. The pixel size was set to 10 nm. For each experiment, 5-10 neurons from 3 different neuronal cultures were imaged, adding to a total number of 15-30 neurons imaged per condition. AnkG immunolabeling was used to identify the AIS and images that covered the full AIS were acquired. The images were deconvolved using Huygens Professional version 22.04 (Scientific Volume Imaging, The Netherlands, http://svi.nl). Hereafter, images were analyzed using the workflow described above, with a grid height of 1 μm and a grid width corresponding to the image width, a frequency range of 170 nm to 230 nm, and an angle range of -20° to +20°. For cross-correlation analysis, an autocorrelation amplitude threshold of 0.6 was applied. Analysis was done with Napari-WaveBreaker v0.2.2 (Vanspauwen, 2023).

#### 2.3.3 Data visualization

Data is represented as either boxplots or violin plots. Within each violin plot, a boxplot is embedded to summarize key statistical metrics. For boxplots, the central line represents the median, the edges of the box correspond to the interquartile range (IQR), and the whiskers extend to 1.5 × IQR. For figures that only contain boxplots, outliers, if present, are shown as individual data points outside the whiskers. Information on statistics is included in each figure's caption.

## 3 Results

### 3.1 Simulated periodicity is accurately reflected by autocorrelation amplitude

To evaluate how well autocorrelation amplitude analysis could estimate periodicity, we generated images with varying periodic localization percentages (percentage of generated points placed within on-line zones, Figures 2A, B). As shown in Figure 2C, the autocorrelation amplitude parameter successfully distinguished between different levels of simulated periodicity, although the sensitivity depended on the periodic localization percentage.

As we aimed to use Napari-WaveBreaker to analyze subtle differences in periodicity in a biological context, we assessed the method's ability to detect small changes by performing statistical comparisons between periodic localization levels differing by only 5%. At periodic localization percentages above 60%, such small increments yielded statistically significant changes in autocorrelation amplitudes (p < 0.05). In contrast, at periodic localization percentages below 60%, distinguishing between groups became more difficult, requiring differences of 10%–15% to achieve statistical significance. These results demonstrate that Napari-WaveBreaker is capable of detecting subtle periodicity changes and is most sensitive when periodic localization exceeds 60%.

### 3.2 Autocorrelation amplitude is mainly influenced by point density

To investigate how image-related parameters influence autocorrelation amplitude, images were generated with variations in point density, kernel size, intensity variation, background noise and pixel size (Table 1, Supplementary Figure S1A). Our analysis revealed that the most impactful parameter was point density, which significantly influenced autocorrelation amplitude in generated images with periodic localization percentages > 60%. In contrast, intensity variation had no noticeable effect. Kernel and pixel size occasionally influenced mean autocorrelation amplitude, but their impact was more pronounced on the IQR (Table 2, Supplementary Figure S1). As expected, the smaller RED kernel, ultimately responsible for creating a sharper image, resulted in a significantly lower autocorrelation amplitude IQR compared to the larger ORANGE kernel (*p* < 0.01). Similarly, images with 100 pixels/μm showed significantly lower autocorrelation IQR than those with 50 pixels/μm (*p* < 0.01). While images with more intensity variation had a slightly higher IQR than those with consistent signal intensities (p < 0.05), this had no effect on the average autocorrelation amplitude. Background noise also influenced autocorrelation amplitude, with the most substantial effects observed at noise levels exceeding 10% (Supplementary Figure S1B).

**Table 2:** Statistical significance of simulation parameters on autocorrelation amplitude and its interquartile range across periodic localization percentages (PLP).

**Analytical metric**	**Point density**	**Kernel size**	**Intensity variation**	**Background noise**	**Pixel size**
**Impact on autocorrelation amplitude**					
PLP: 50%	ns	ns	ns	***	ns
PLP: 55%	ns	ns	ns	***	ns
PLP: 60%	***	ns	ns	*	ns
PLP: 65%	***	**	ns	*	ns
PLP: 70%	***	ns	ns	*	ns
PLP: 75%	***	ns	ns	ns	ns
PLP: 80%	**	ns	ns	*	*
PLP: 85%	**	ns	ns	**	**
PLP: 90%	***	ns	ns	*	ns
PLP: 95%	***	ns	ns	ns	**
PLP: 100%	***	ns	ns	ns	ns
Interquartile range	ns	**	*	*	**

ns, not significant, *p < 0.05, **p < 0.01, *** *p* < 0.001.

### 3.3 Cross-correlation analysis accurately distinguishes between alternating and overlapping patterns

**Figure 3:**
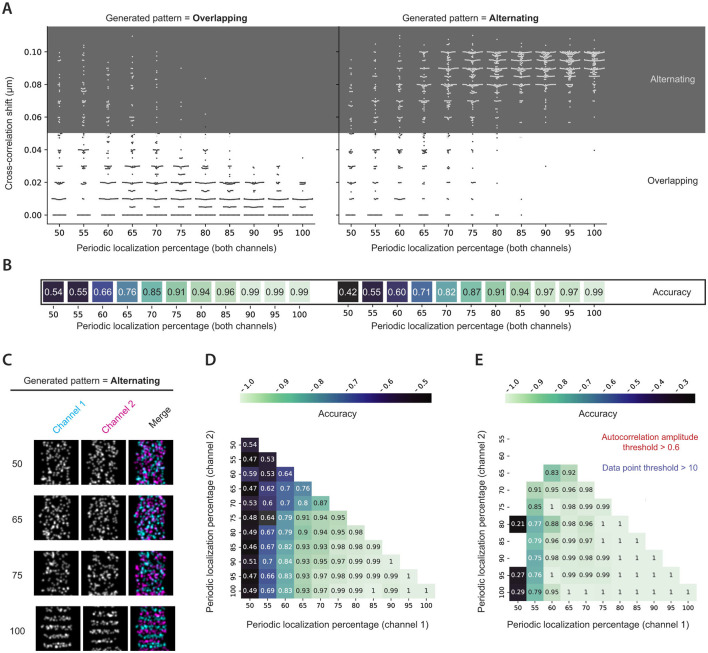
Cross-correlation analysis of simulated images. **(A)** Cross-correlation shifts calculated for generated two-channel images with the indicated periodic localization percentages. Both channels were generated with the same periodic localization percentage. The analysis was performed for both overlapping and alternating simulated patterns. White and gray zones indicate whether the cross-correlation shift was classified as overlapping or alternating. **(B)** Cross-correlation accuracies for the indicated periodic localization percentages in **(A)**. **(C)** Examples of grids with an alternating interdistance between channels for the indicated periodic localization percentages. **(D)** Cross-correlation accuracy achieved for all possible periodic localization percentage combinations in the two-channel simulated images. **(E)** Data from D subjected to a 0.6 autocorrelation amplitude threshold and a minimum data point criterion of 10. Besides a variable periodic localization percentage, simulations were performed with default parameters.

To validate cross-correlation analysis, two-channel images were generated, both with overlapping and alternating periodicity between the channels (Figure 3C). The analysis then determined the cross-correlation shift, which could classify a grid into either an overlapping or alternating category. The ability to predict whether a generated pattern was overlapping or alternating will henceforth be referred to as accuracy. As expected, for both overlapping and alternating generated patterns, the accuracy increased gradually as the periodic localization percentage of the individual channels rose, and the calculated cross-correlation shift was more likely to fall into the correct pattern zone (Figure 3A). For both overlapping and alternating generated patterns, accuracy exceeded 70% at a periodic localization fraction of only 65%. Interestingly, when comparing the method's accuracy between periodic localization percentages, the analysis of overlapping patterns often resulted in slightly higher accuracy than that of alternating patterns (Figure 3B). Furthermore, when comparing the accuracy of the cross-correlation approach when analyzing two-channel images where the channels were generated with different periodic localization percentages, it was evident that the accuracy was largely dependent on the channel with the lowest percentage (Figure 3D).

While the cross-correlation analysis displayed good accuracy, it could be further improved by considering the calculated autocorrelation amplitude for both channels. When plotting accuracy for various periodic localization percentages and splitting the data points based on the minimum autocorrelation amplitude of both channels, it became clear that much of the poor cross-correlation accuracy originated from data points with low autocorrelation amplitude (Supplementary Figure S2). Additionally, the remaining bins with low accuracy often had a limited number of values on which the accuracy was based (bins with fewer than 10 data points highlighted with red-bordered boxes in Supplementary Figure S2). The analysis suggested that implementing a minimum autocorrelation amplitude threshold of 0.6 and a minimum data point criterion of 10 could dramatically improve the achieved accuracy, which was confirmed by applying these two considerations to the complete analysis of simulated images (Figure 3E).

### 3.4 Autocorrelation amplitude reflects the visual periodicity level of STED images

**Figure 4:**
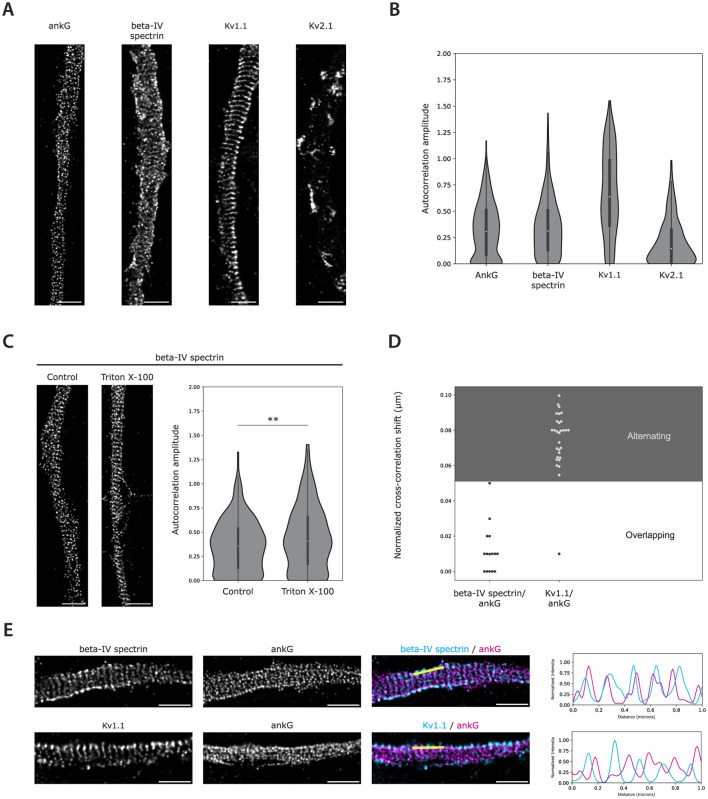
Auto- and cross-correlation analysis validation on STED images. **(A)** Deconvolved STED images of AISs of 22 DIV cultured rat hippocampal neurons immunolabeled for ankG, beta-IV spectrin, Kv1.1, and Kv2.1. **(B)** Autocorrelation amplitudes for the indicated AIS proteins. Number of grids analyzed: ankG = 1,045, beta-IV spectrin = 437, Kv1.1 = 333, Kv2.1 = 275. **(C)** Deconvolved STED images and autocorrelation amplitudes of AISs immunolabeled for beta-IV spectrin in neurons with and without 0.5% Triton X-100 extraction prior to fixation. Number of grids analyzed: control = 344, Triton X-100 = 474. Statistical analysis was performed using the Mann-Whitney U test (**p-value < 0.01). **(D)** Normalized cross-correlation shifts obtained from two-channel images of ankG in combination with beta-IV spectrin or Kv1.1 as shown in **(E)**. The alternating (gray) and overlapping (white) zones are indicated. Data were subjected to a 0.6 minimum autocorrelation amplitude threshold. Number of grids included after thresholding: beta-IV spectrin = 15, Kv1.1 = 30. **(E)** Two-channel images of beta-IV spectrin and Kv1.1 in combination with ankG. Normalized fluorescence intensity profiles for both channels along the 1 μm yellow lines are plotted to the right. Scale bars: 1 μm.

After validating the method on simulated data, we applied the analysis to STED images of known periodic and non-periodic AIS-localized targets in cultured rat hippocampal neurons (Figure 4). Autocorrelation amplitude accurately reflected the mild periodicity of ankG and beta-IV spectrin (C-terminus) achieved in our experimental setup with an average autocorrelation amplitude of 0.33 (IQR: 0.09–0.51) and 0.35 (IQR: 0.13–0.50) respectively (Figures 4A, B). In addition, the voltage-gated potassium channel Kv2.1, which is localized in unique AIS clusters without any apparent periodicity (King et al., 2014), displayed a much lower average autocorrelation amplitude of 0.20 (IQR 0.02–0.32). In contrast, the voltage-gated potassium channel Kv1.1, like its paralog Kv1.2 (D'Este et al., 2017), exhibited a remarkable periodic localization pattern, which was reflected by an average autocorrelation amplitude of 0.66 (IQR: 0.36-0.98) (Figures 4A, B).

To further investigate whether autocorrelation amplitude could determine slight alterations in periodicity, hippocampal neurons were treated with 0.5% Triton X-100 before fixation and immunolabeled with antibodies directed against the C-terminus of beta-IV spectrin. The goal was to investigate whether Triton X-100 detergent extraction would improve the periodicity, as the AIS MPS core and its associated proteins are resistant to this treatment (Torii et al., 2020). Visual inspection of acquired STED images suggested that the beta-IV spectrin immunolabeling displayed a stronger periodic pattern after Triton X-100 extraction (Figure 4C). Indeed, this observation was reflected by a significant increase in the calculated autocorrelation amplitude of TX-100 extracted cells (autocorrelation amplitude: 0.44, IQR: 0.17-0.66) as compared to control cells (autocorrelation amplitude: 0.36, IQR: 0.13–0.54, **p < 0.01) (Figure 4C).

### 3.5 Cross-correlation analysis reliably detects periodic co- and alternating distribution of AIS localized proteins

To determine whether the cross-correlation-based workflow could reliably identify relative shifts between periodically distributed AIS targets, we captured STED images of cultured hippocampal neurons co-immunolabeled for ankG and beta-IV spectrin (C-terminus). Visual inspection of the images suggested an overlap between the beta-IV spectrin and ankG immunolabelings, which was further supported by the fluorescence intensity profiles (Figure 4E). In line with this observation, the cross-correlation shift values were all located in the zone considered overlapping (100% of points, Figure 4D), which is in accordance with previous literature (Leterrier et al., 2015).

We then applied the same analysis to STED images of neurons co-immunolabeled for Kv1.1 and ankG. The Kv1 complex has been reported to co-localize with the actin rings and would be expected to alternate with the localization of ankG (D'Este et al., 2017). Indeed, visual inspection of the images suggested an alternating localization pattern between the two AIS proteins, which was supported by the fluorescence intensity profiles (Figure 4E). Furthermore, cross-correlation analysis confirmed this observation as 97% of the cross-correlation shift values revealed an alternating localization pattern (Figure 4D).

## 4 Discussion

In this paper, we present an automated method for quantifying MPS-associated periodicity and evaluating the spatial shift between two MPS-associated periodic targets. We show that the method can detect small changes in periodicity in both simulated and STED microscopy images. We furthermore show that the embedded cross-correlation analysis can accurately determine whether two periodically localized targets display an overlapping or alternating localization pattern, even at low periodicity levels.

The strength of the method lies in the combination of a non-biased region of interest selection and the flexibility of the correlation functions used. It allows for the analysis of whole images, thereby applying non-selective criteria for defining the regions to be analyzed. The implementation of the analysis of periodicity in all angles was a key feature in fulfilling this goal. This unselective approach improves on previous strategies which did rely on correlation-based analysis, however, applied to manually selected regions (Zhong et al., 2014; Albrecht et al., 2016; D'Este et al., 2017; Wang et al., 2019; Fréal et al., 2023). We decided to use autocorrelation since the output values are less affected by interpeak intensity variation and background noise compared to sinusoidal curve fitting (Leterrier et al., 2015). We furthermore decided that treating periodicity like a range was more appropriate than the binary choice often applied. This facilitates the evaluation of changes in periodicity between, for example, wild type and mutated targets or treated and non-treated samples. Indeed, the Napari-WaveBreaker plugin has already been used to quantify the periodic localization of the AIS ion channel TRAAK and to determine its nanoscale co-localization with ankG (Luque-Fernández et al., 2024). In addition, the analysis is not bound to one exact frequency value as it is in the Pearson correlation approach of Barabas et al. (2017), but rather a frequency range of choice or a completely unrestricted range if required. This feature facilitates analysis in samples with variations in MPS frequency as well as the study of disrupted periodic patterns. Among others, this could benefit the analysis of periodic patterns in samples that have been immunolabeled in expansion microscopy techniques (Martínez et al., 2020), where the case-to-case expansion factor proportionally affects the inter-peak distance.

We also examined which image parameters should be considered in the analysis. While it is better established that experimental conditions should be the same (e.g., same fluorophore and microscope settings for comparisons), we observed that other factors that are inherent to the samples and/or the staining procedure should also be considered. Our simulations demonstrated that the point density of the samples is the most important parameter (Table 2), which should be considered if it varies greatly between experimental groups. Along the same lines, we do not recommend direct statistical comparisons of autocorrelation amplitudes for targets immunolabeled with different antibodies.

We finally demonstrated the tool's ability to determine whether two periodically localized targets overlap or alternate in their distributions. By calculating their spatial shift by cross-correlation, we were able to demonstrate that while the beta-IV spectrin C-terminus overlaps with the localization of ankG, Kv1.1 alternates with it, which aligns with previously observed Kv1.2 colocalization with actin rings (D'Este et al., 2017). While cross-correlation has been utilized to show that two periodically localized targets overlap or alternate (Xu et al., 2013; Albrecht et al., 2016; D'Este et al., 2017; Zhou et al., 2022), it was never used to calculate the interdistance between targets in unbiasedly selected regions. Our simulations show that applying an autocorrelation amplitude threshold in the analysis improves cross-correlation shift accuracy. In our case, the applied threshold was 0.6, as suggested by our simulated data analysis. Nonetheless, when choosing the size of the threshold, it should be considered how many data points are lost in the process, as too few data points can generate misleading results.

In conclusion, we here developed a method to quantify target periodicity and two target interdistances in superresolution microscopy images. The developed method is available as a tool, Napari-WaveBreaker, an open-source plugin for the interactive image viewer Napari. It thus represents a widely accessible method for unbiased quantifications of MPS-associated periodicity and its changes. While this study validates the method using STED images, its design makes it well-suited for broader use, including with STORM and emerging super-resolution techniques such as expansion microscopy, highlighting its potential for wide applicability in the field.

## Data Availability

The datasets presented in this study can be found in online repositories. The names of the repository/repositories and accession number(s) can be found below: osf.io/h3pja.
