# Supplementary material for: A correlation-based tool for quantifying membrane periodic skeleton associated periodicity
Source: Front Neuroinform. 2025 Aug 22;19:1628538. doi: 10.3389/fninf.2025.1628538 (PMC12411523; doi:10.3389/fninf.2025.1628538)
Supplement: Supplementary file 1 [file Supplementary_file_1.pdf]

frontiers

Supplementary Material

1 SUPPLEMENTARY TABLES AND FIGURES

1.1 Figures

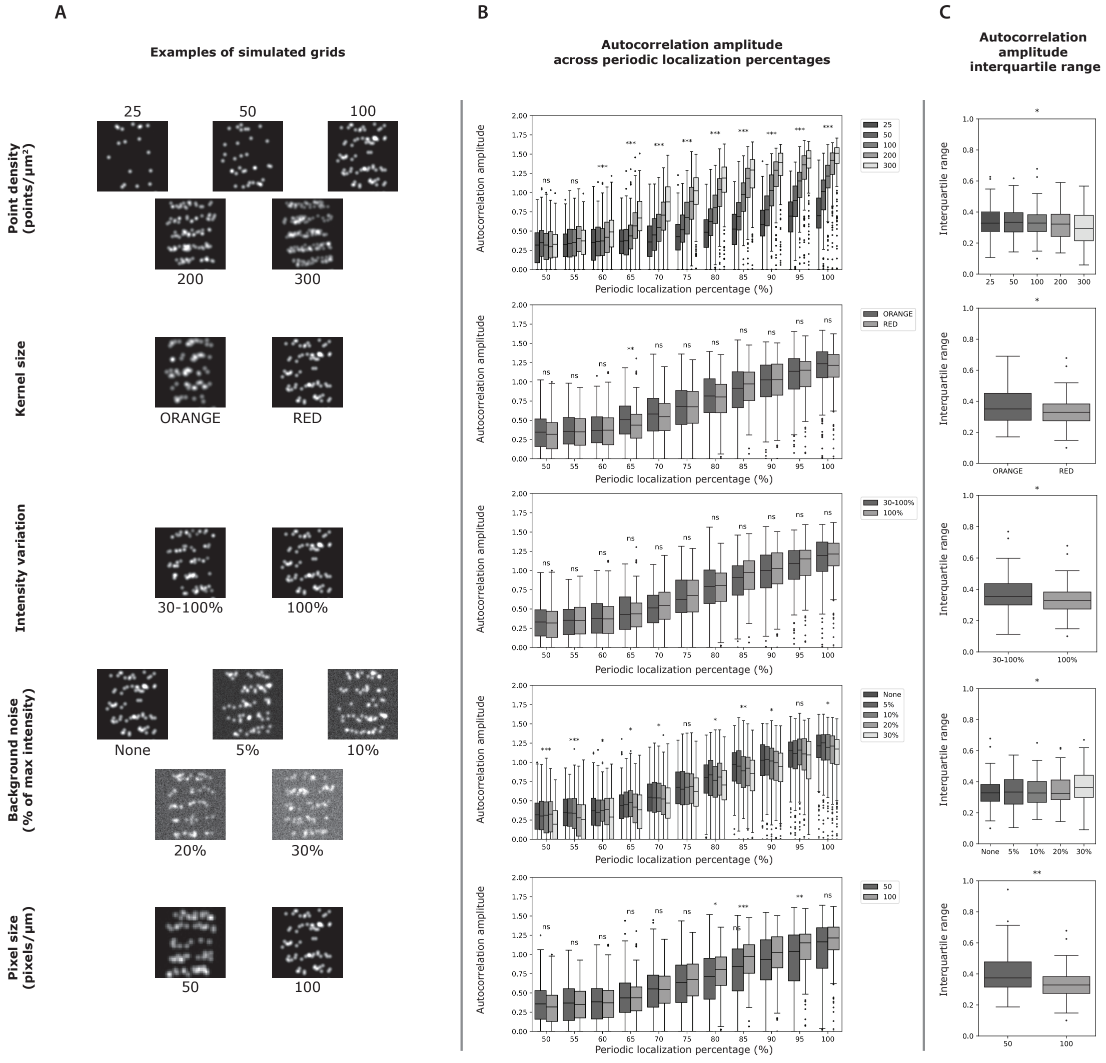

Figure S1. Influence of image-related parameters on autocorrelation amplitude. **(A)** Example of the same grid simulated with various variable parameters. For each analyzed variable, all other simulation parameters were held at nominal values. In these examples, the periodic localization percentage of the simulated images was 100% **(B)** Distribution of autocorrelation amplitude across all periodic localization percentages for each analyzed parameter. The statistical significance of each parameter's influence at each periodic localization percentage was calculated using a Kruskal-Wallis test for point density and background noise and a Mann-Whitney U test for all other parameters. **(C)** Distribution of per-image interquartile range of autocorrelation amplitude for all analyzed parameters. Statistical significance was determined using one-way ANOVA for point density and background noise and a Student's t-test for all other parameters. Significance levels: *** for p-value < 0.001, ** for p-value < 0.01, * for p-value < 0.05, and "ns" for non-significant.

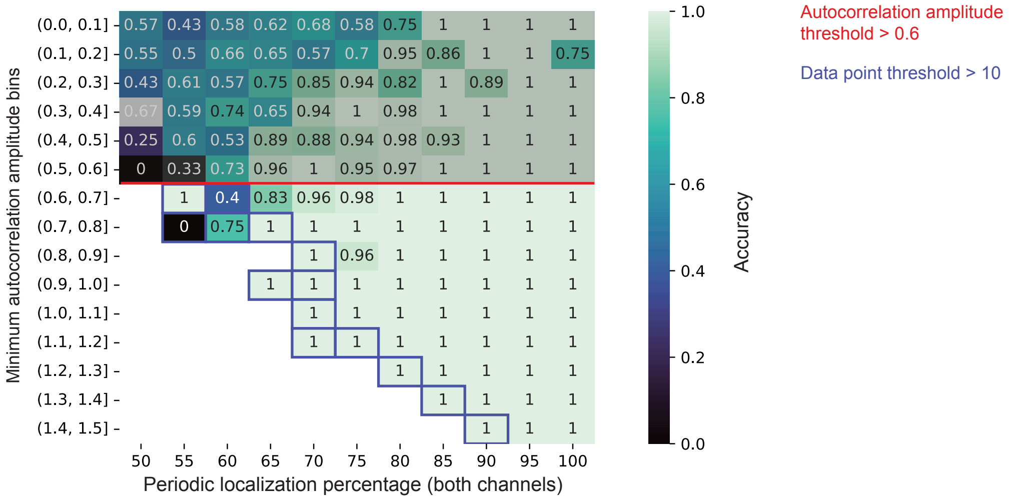

Figure S2. Influence of autocorrelation amplitude on cross-correlation accuracy. Heat map of cross-correlation accuracy per periodic localization percentage, distributed over segmented bins of the minimum autocorrelation amplitude of both channels of the analyzed grid. The red horizontal line indicates a 0.6 autocorrelation amplitude threshold. Red-bordered boxes highlight data points based on fewer than 10 data points. Besides the variable periodic localization percentage, simulations were performed with nominal parameters.
